# Supplementary material for: Expression of tardigrade intrinsically disordered proteins in C. elegans reveals variable effects on aging and stress
Source: bioRxiv. 2026 Oct 31:2026.10.26.762026. Preprint. [Version 1] doi: 10.64898/2026.10.26.762026 (PMC13733966; doi:10.64898/2026.10.26.762026)
Supplement: Supplementary file 1 [file NIHPP2026.10.26.762026V1-supplement-1.pdf]

bioRxiv preprint doi: https://doi.org/10.64898/2026.10.26.762026; this version posted October 31, 2026. The copyright holder for this preprint (which was not certified by peer review) is the author/funder, who has granted bioRxiv a license to display the preprint in perpetuity. It is made available under a CC-BY 4.0 International license.

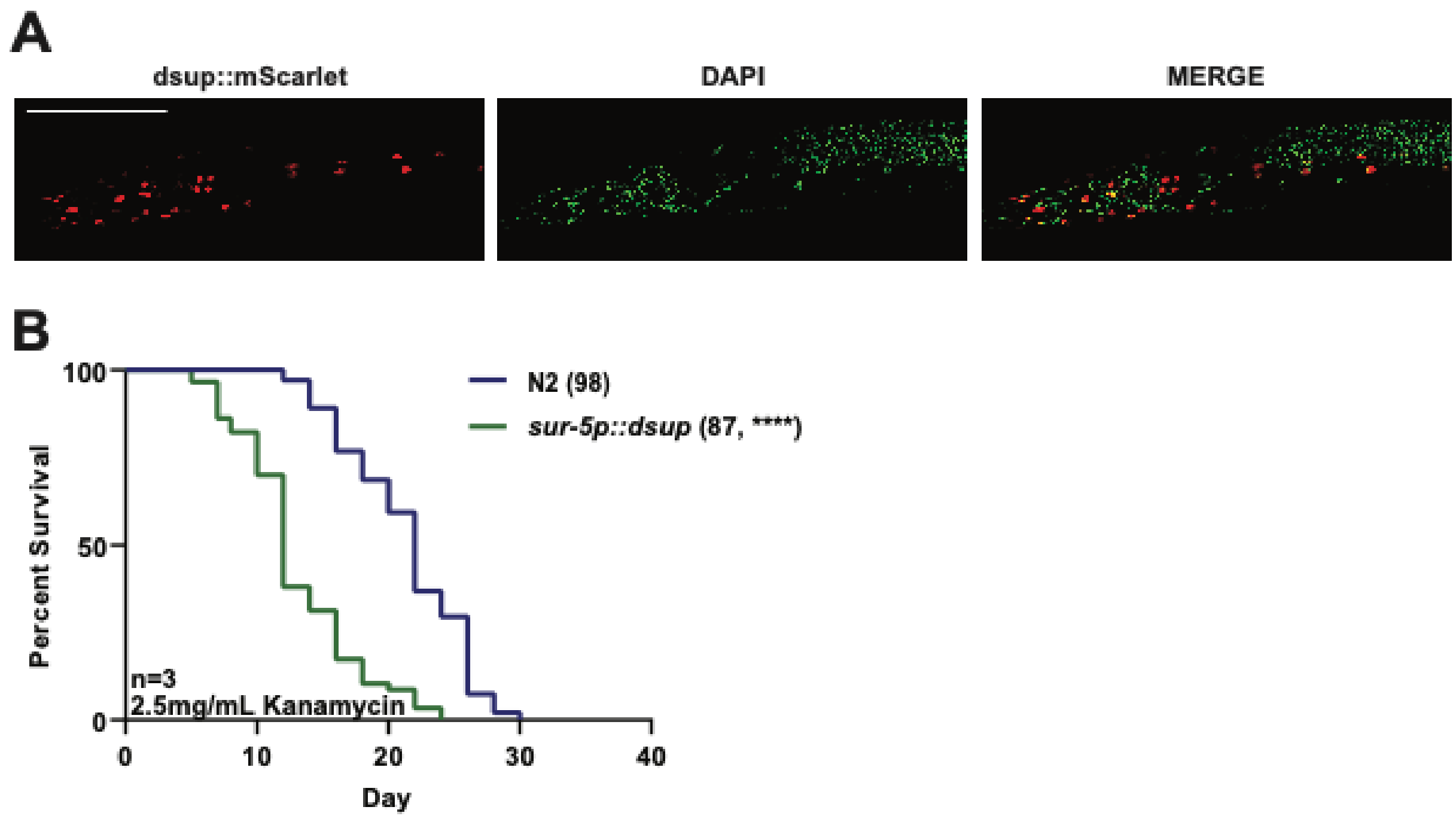

Fig. S1. Somatic DSUP localizes to nuclei and reduces lifespan independently of bacterial pathogenesis. (A) Representative fluorescent images of animals expressing DSUP in somatic tissues (*sur-5p::dsup*). DSUP fluorescence is shown in red, DAPI nuclear staining in green, and the merged image demonstrates nuclear localization of DSUP. Scale bar is 100 µm. **(B)** Lifespan of wild-type N2 and *sur-5p::dsup* animals maintained on kanamycin-killed bacteria. Numbers in parentheses indicate total animals scored. Statistical significance was determined by Log-Rank testing. Statistical information for all replicates is available in **Table S2**. Note: the lifespan data was collected and analyzed together with data from **Fig. S2D**. **** = $p < 0.0001$.

bioRxiv preprint doi: https://doi.org/10.64898/2026.10.26.762026; this version posted October 31, 2026. The copyright holder for this preprint (which was not certified by peer review) is the author/funder, who has granted bioRxiv a license to display the preprint in perpetuity. It is made available under a CC-BY 4.0 International license.

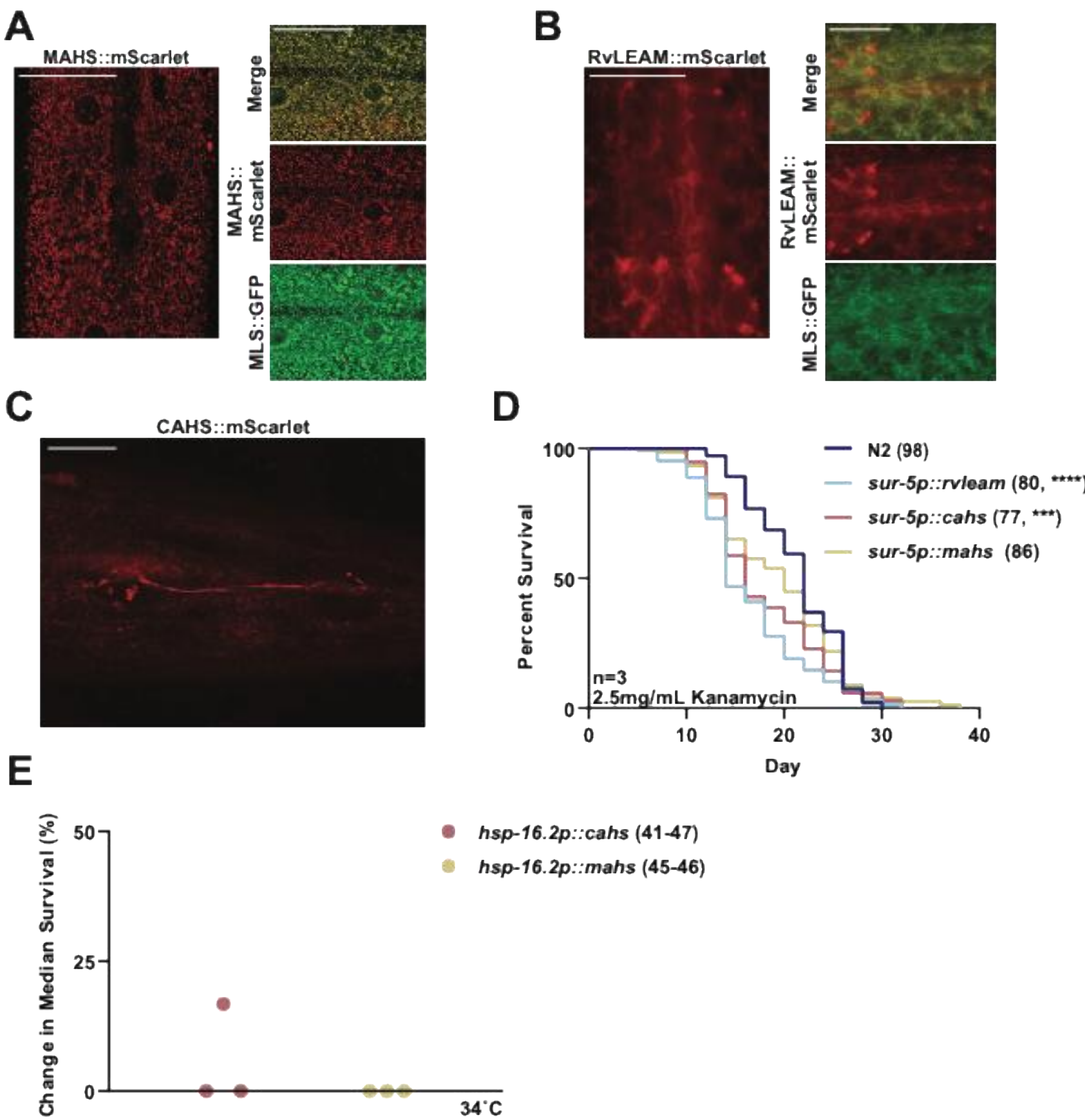

Fig. S2. Subcellular localization of RvLEAM, CAHS, and MAHS in *C. elegans*. (A) Representative fluorescent images of animals expressing MAHS::mScarlet shown in red, mitochondrial matrix-localized GFP (MLS::GFP) in green, and the merged image demonstrates colocalization of MAHS with mitochondria. Scale bar is 20 µm. **(B)** Representative fluorescent images of animals expressing RvLEAM::mScarlet shown in red, MLS::GFP in green, and the merged image demonstrates predominant mitochondrial localization of RvLEAM. Scale bar is 10 µm. **(C)** Representative fluorescent image of animals expressing CAHS::mScarlet, demonstrating diffuse cytoplasmic localization. Scale bar is 20 µm. **(D)** Lifespan of wild-type N2 animals and transgenic animals constitutively expressing RvLEAM (*sur-5p::rvleam*), CAHS (*sur-5p::cahs*), or MAHS (*sur-5p::mahs*) in somatic tissues. Animals are grown on bacteria treated

bioRxiv preprint doi: https://doi.org/10.64898/2026.10.26.762026; this version posted October 31, 2026. The copyright holder for this preprint (which was not certified by peer review) is the author/funder, who has granted bioRxiv a license to display the preprint in perpetuity. It is made available under a CC-BY 4.0 International license.

with 2.5 mg/mL kanamycin. Numbers in parentheses indicate total animals scored. Statistical significance was determined by Log-Rank testing. Statistical information for all replicates is available in **Table S2**. Note: the lifespan data was collected and analyzed together with data from **Fig. S1B. (E)** Change in median survival during thermotolerance assays of animals expressing CAHS or MAHS under the heat-shock-inducible *hsp-16.2* promoter. Animals were exposed to 34°C, and each point represents one independent biological replicate. N=34-47. *** = $p < 0.001$; **** = $p < 0.0001$; ns = not significant, $p > 0.05$.

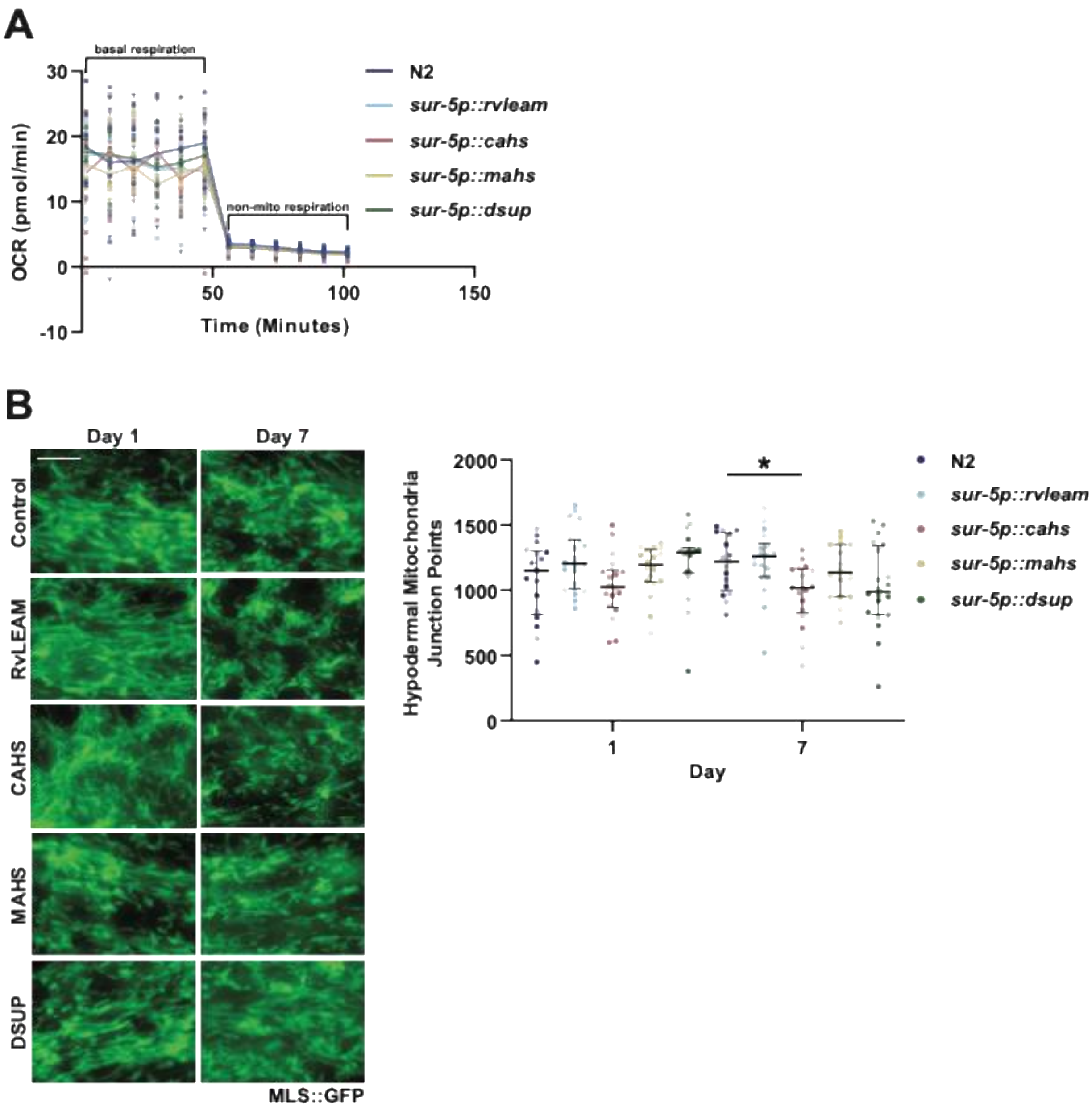

Fig. S3. Somatic RvLEAM, CAHS, MAHS or DSUP expression does not significantly alter mitochondrial respiration, and only CAHS expression affects mitochondrial morphology in the hypodermis. (A) Basal oxygen consumption rate (OCR) of wild-type N2, *sur-5p::rvleam*,

bioRxiv preprint doi: https://doi.org/10.64898/2026.10.26.762026; this version posted October 31, 2026. The copyright holder for this preprint (which was not certified by peer review) is the author/funder, who has granted bioRxiv a license to display the preprint in perpetuity. It is made available under a CC-BY 4.0 International license.

sur-5p::cahs, *sur-5p::mahs*, and *sur-5p::dsup* animals. OCR was measured using a Seahorse XFe96 Analyzer and normalized to the number of worms per well. Sodium azide was added to determine non-mitochondrial respiration. Data is presented as a SuperPlot with each individual dot representing one animal, different shades indicating each independent biological replicate, and line indicating the median at all time points. **(B)** Right: Representative fluorescent images of hypodermal mitochondrial morphology (*col-19p::MLS::GFP*) at day 1 and day 7 in wild-type N2, *sur-5p::rvleam* (RvLEAM), *sur-5p::cahs* (CAHS), *sur-5p::mahs* (MAHS), and *sur-5p::dsup* (DSUP) animals. Scale bar is 5µm. Left: Quantification of images using mitoMAPR. Mitochondrial morphology was quantified by the number of mitochondrial junction points. Data is presented as a SuperPlot with each individual dot representing one animal, different shades indicating each independent biological replicate, and lines indicating the median and interquartile range. * = $p < 0.05$ using non-parametric Mann-Whitney testing. All raw mitoMAPR analysis is available in **Table S3**.

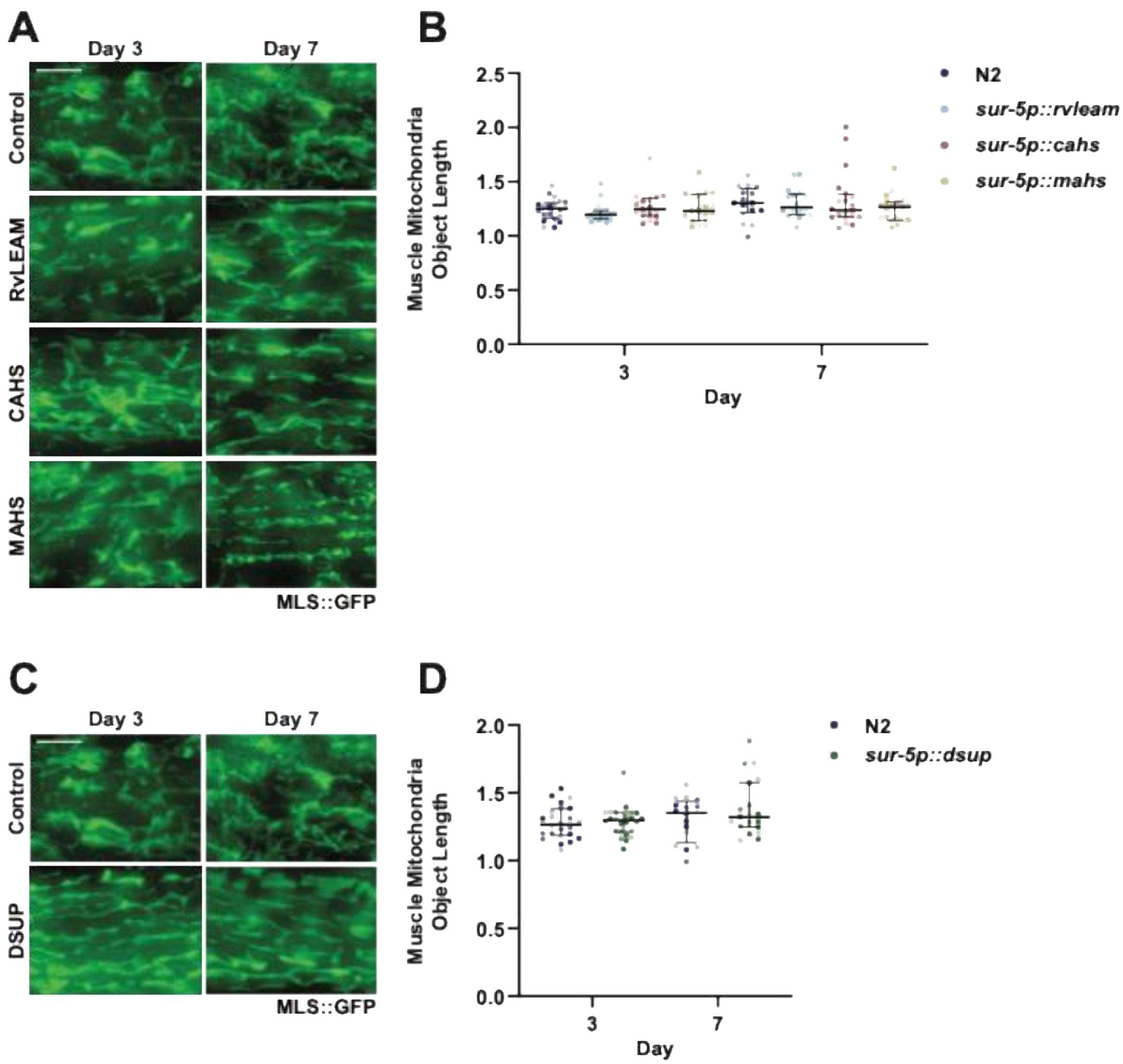

bioRxiv preprint doi: https://doi.org/10.64898/2026.10.26.762026; this version posted October 31, 2026. The copyright holder for this preprint (which was not certified by peer review) is the author/funder, who has granted bioRxiv a license to display the preprint in perpetuity. It is made available under a CC-BY 4.0 International license.

Fig. S4. Somatic RvLEAM, CAHS, MAHS or DSUP expression does not significantly alter mitochondrial morphology in the muscle. (A) Representative fluorescent images of muscle mitochondrial morphology (*myo-3p::MLS::GFP*) at day 1 and day 7 in wild-type N2, *sur-5p::rvleam* (RvLEAM), *sur-5p::cahs* (CAHS), and *sur-5p::mahs* (MAHS). Scale bar is 5μm. **(B)** Quantification of images using mitoMAPR. Mitochondrial morphology was quantified by mitochondrial object length. Data is presented as a SuperPlot with each individual dot representing one animal, different shades indicating each independent biological replicate, and lines indicating the median and interquartile range. Note: two of three replicates were collected and analyzed together with data from **Fig. S4D**. **(C)** Representative fluorescent images of muscle mitochondrial morphology (*myo-3p::MLS::GFP*) at day 1 and day 7 in wild-type N2 and *sur-5p::dsup* (DSUP) animals. Scale bar is 5μm. **(D)** Quantification of images using mitoMAPR. Mitochondrial morphology was quantified by mitochondrial object length. Data is presented as a SuperPlot with each individual dot representing one animal, different shades indicating each independent biological replicate, and lines indicating the median and interquartile range. All statistical testing were not significant using non-parametric Mann-Whitney testing. All raw mitoMAPR analysis is available in **Table S3**. Note: two of three replicates were collected and analyzed together with data from **Fig. S4B**.

bioRxiv preprint doi: https://doi.org/10.64898/2026.10.26.762026; this version posted October 31, 2026. The copyright holder for this preprint (which was not certified by peer review) is the author/funder, who has granted bioRxiv a license to display the preprint in perpetuity. It is made available under a CC-BY 4.0 International license.

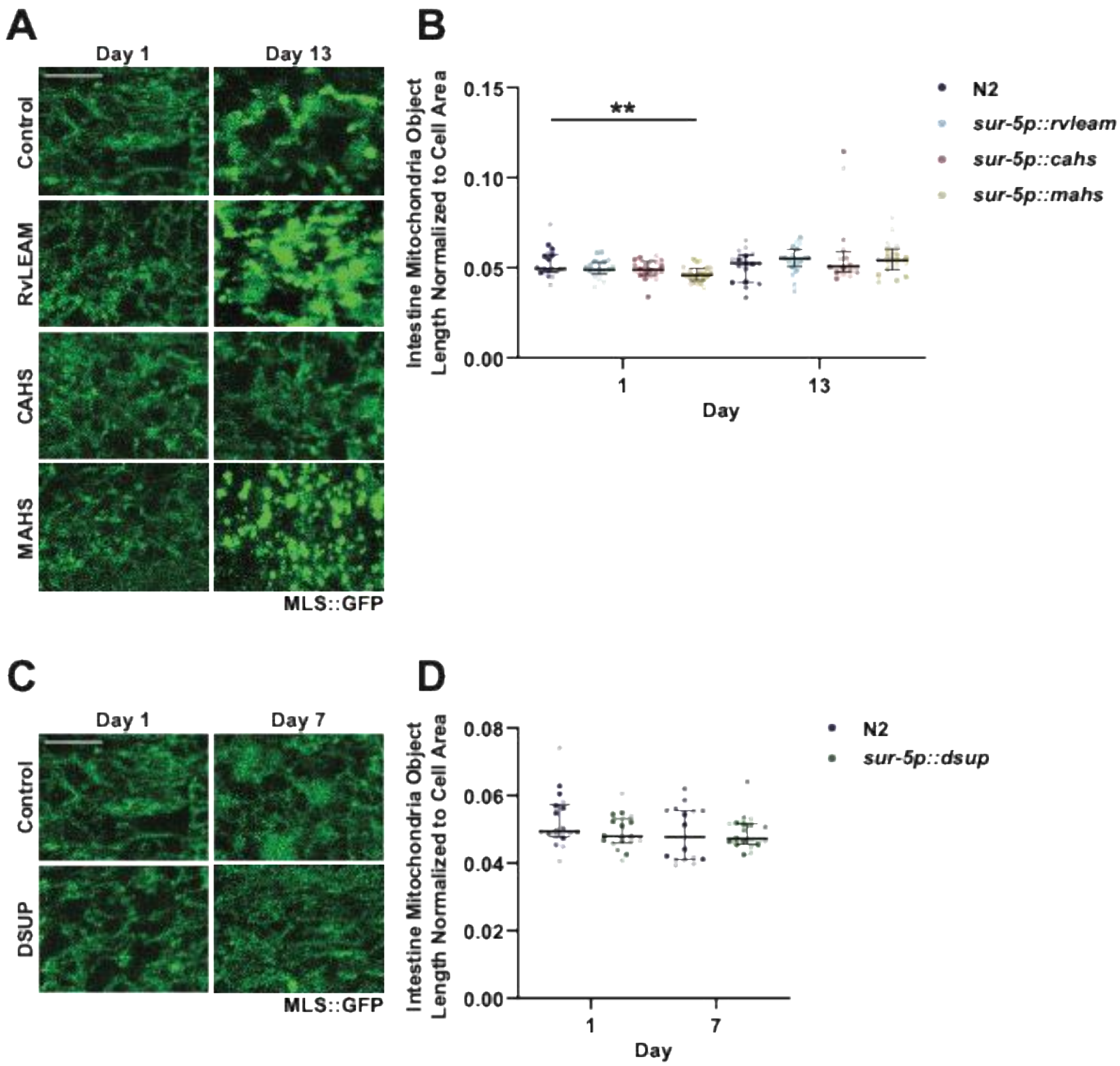

Fig. S5. Somatic RvLEAM, CAHS, or DSUP expression does not significantly alter mitochondrial morphology, while MAHS has subtle, but statistically significant changes in the intestine. (A) Representative fluorescent images of intestinal mitochondrial morphology (*vha-6p::MLS::GFP*) at day 1 and day 13 in wild-type N2, *sur-5p::rvleam* (RvLEAM), *sur-5p::cahs* (CAHS), and *sur-5p::mahs* (MAHS) animals. Scale bar is 5μm. **(B)** Quantification of (A) using mitoMAPR. Mitochondrial morphology was quantified by mitochondrial object length normalized to the cell area. Data is presented as a SuperPlot with each individual dot representing one animal, different shades indicating each independent biological replicate, and lines indicating the median and interquartile range**.** Note: the day 1 data was collected and analyzed together with data from **Fig. S5D**. **(C)** Representative fluorescent images of intestinal mitochondrial morphology (*vha-6p::MLS::GFP*) at day 1 and day 7 in wild-type N2 and *sur-5p::dsup* (DSUP) animals. Scale bar is 5μm. **(D)** Quantification of (C) using mitoMAPR. Mitochondrial morphology was quantified by mitochondrial object length normalized to the cell area. Data is presented as a SuperPlot with each individual dot representing one animal, different shades indicating each independent biological replicate, and lines indicating the

bioRxiv preprint doi: https://doi.org/10.64898/2026.10.26.762026; this version posted October 31, 2026. The copyright holder for this preprint (which was not certified by peer review) is the author/funder, who has granted bioRxiv a license to display the preprint in perpetuity. It is made available under a CC-BY 4.0 International license.

median and interquartile range**.** All statistical testing were not significant using non-parametric Mann-Whitney testing. All raw mitoMAPR analysis is available in **Table S3**. Note: the day 1 data was collected and analyzed together with data from **Fig. S5B**.

bioRxiv preprint doi: https://doi.org/10.64898/2026.10.26.762026; this version posted October 31, 2026. The copyright holder for this preprint (which was not certified by peer review) is the author/funder, who has granted bioRxiv a license to display the preprint in perpetuity. It is made available under a CC-BY 4.0 International license.

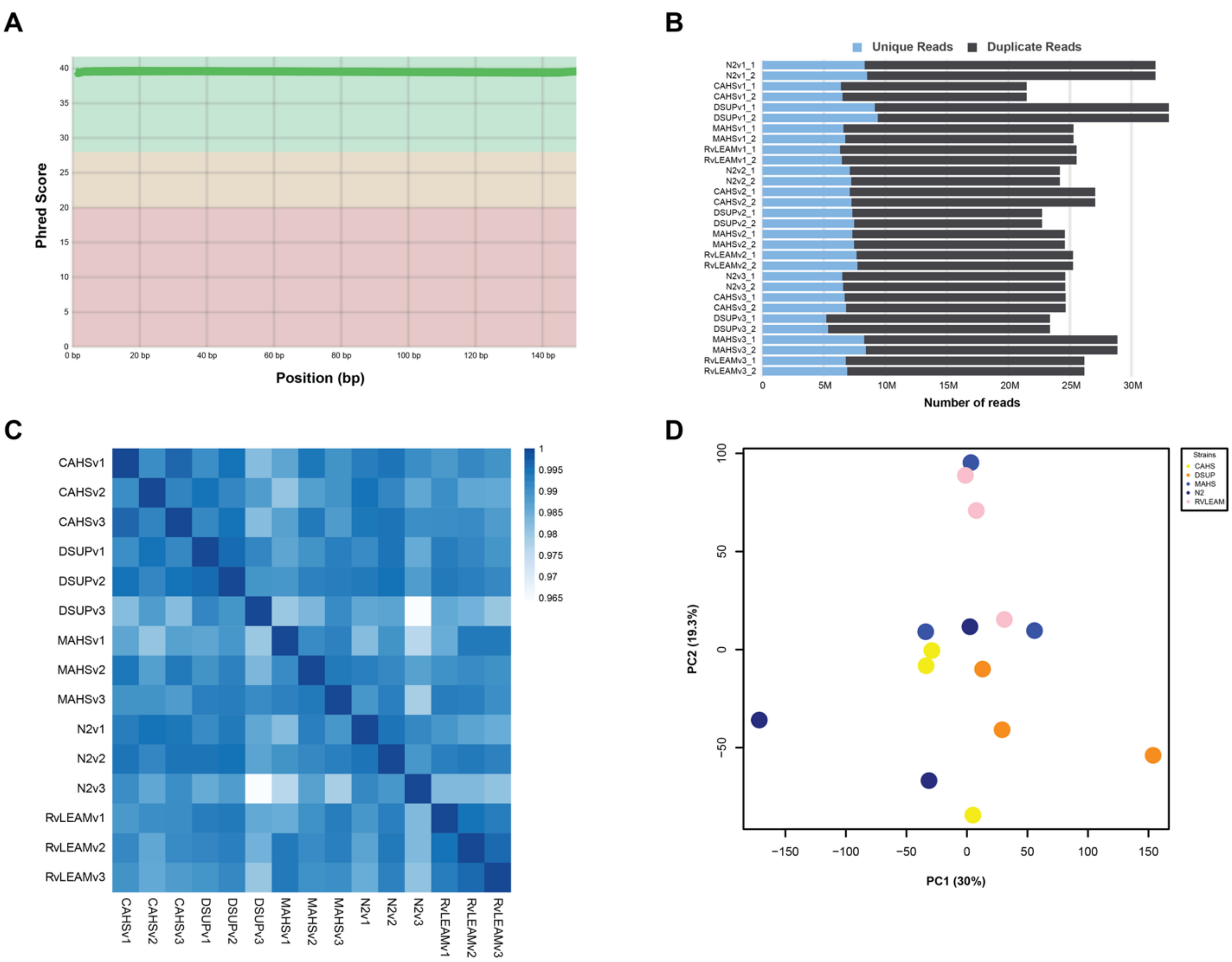

bioRxiv preprint doi: https://doi.org/10.64898/2026.10.26.762026; this version posted October 31, 2026. The copyright holder for this preprint (which was not certified by peer review) is the author/funder, who has granted bioRxiv a license to display the preprint in perpetuity. It is made available under a CC-BY 4.0 International license.

Fig. S6. RNA-seq quality control and sample assessment for TDP-expressing C. elegans strains. (A) Per-base sequence quality scores across reads, showing consistently high Phred scores across all nucleotide positions. **(B)** Number of unique and duplicate reads for each biological replicate of N2, CAHS, DSUP, MAHS, and RvLEAM samples. **(C)** Correlation heatmap showing pairwise similarity among RNA-seq samples, with biological replicates generally exhibiting high correlation. **(D)** Principal component analysis (PCA) of RNA-seq samples, with points colored by strain. PC1 and PC2 explain 30% and 19.3% of the total variance, respectively, and illustrate variation both within and between strain groups.

bioRxiv preprint doi: https://doi.org/10.64898/2026.10.26.762026; this version posted October 31, 2026. The copyright holder for this preprint (which was not certified by peer review) is the author/funder, who has granted bioRxiv a license to display the preprint in perpetuity. It is made available under a CC-BY 4.0 International license.

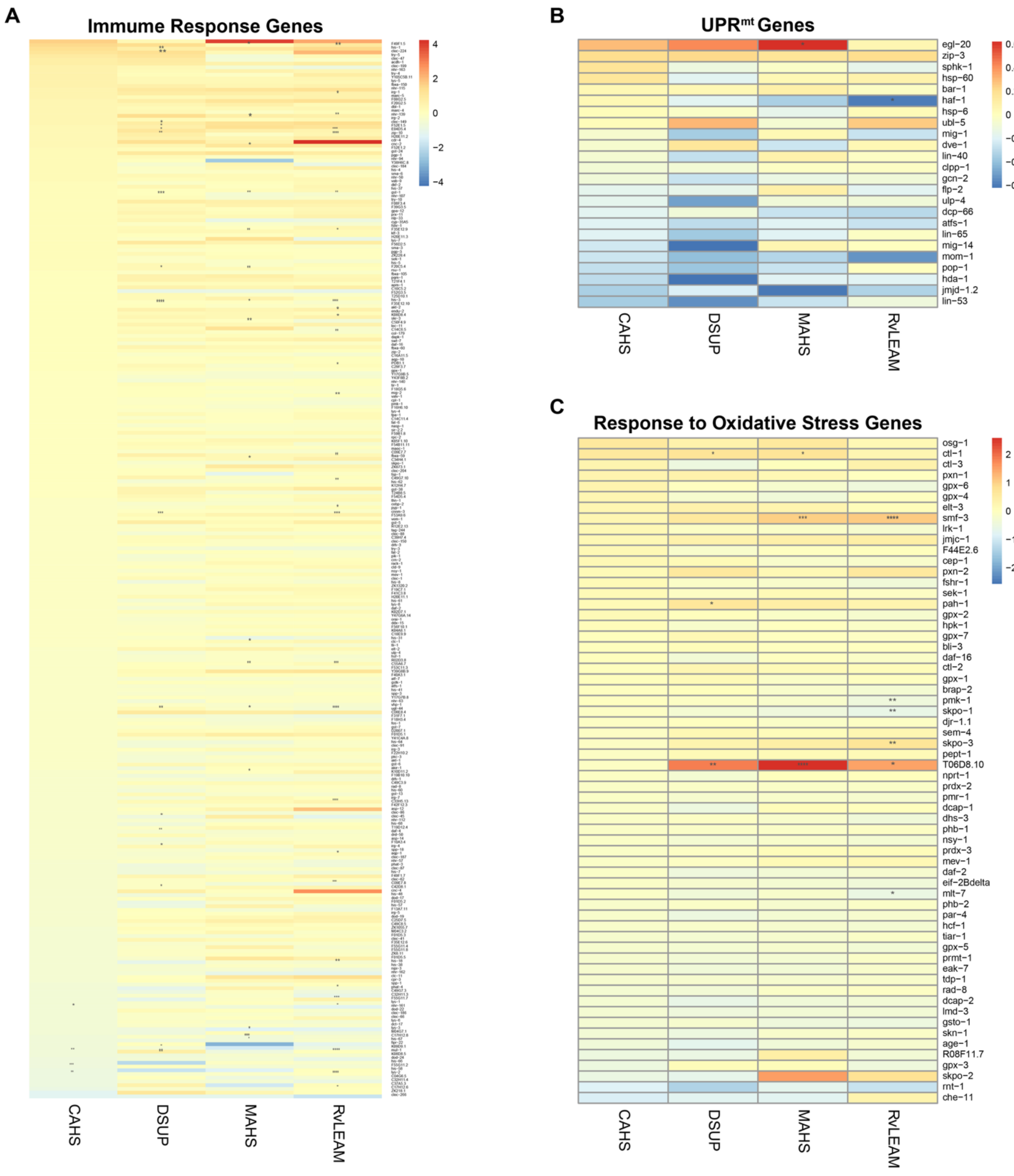

Fig. S7. Tardigrade-derived proteins alter expression of stress-response and immune-related genes. (A) Heatmap showing differential expression of immune response genes (GO:0006955) in animals expressing CAHS, DSUP, MAHS, or RvLEAM relative to control animals. **(B)** Heatmap showing differential expression of genes associated with the mitochondrial unfolded protein response (GO:0034514) across the four TDP-expressing strains. **(C)** Heatmap showing differential expression of genes associated with the response to oxidative stress (GO:0006979). Colors indicate the magnitude and direction of gene expression changes, with red representing increased expression and blue representing decreased expression

bioRxiv preprint doi: https://doi.org/10.64898/2026.10.26.762026; this version posted October 31, 2026. The copyright holder for this preprint (which was not certified by peer review) is the author/funder, who has granted bioRxiv a license to display the preprint in perpetuity. It is made available under a CC-BY 4.0 International license.

relative to control. Asterisks indicate statistically significant differential expression. All differentially expressed genes for RNA-seq experiments are available in **Table S4** and genes used to construct heat-maps are available in **Table S5**.
